# Cognitive Representations and Cognitive Processing of Team-Specific Tactics in Soccer

**DOI:** 10.1371/journal.pone.0118219

**Published:** 2015-02-25

**Authors:** Heiko Lex, Kai Essig, Andreas Knoblauch, Thomas Schack

**Affiliations:** 1 Bielefeld University, Research Group Neurocognition and Action—Biomechanics, Bielefeld, Germany; 2 Bielefeld University, Cognitive Interaction Technology—Center of Excellence, Bielefeld, Germany; 3 Albstadt-Sigmaringen University, Engineering Faculty, Albstadt-Ebingen, Germany; University of Granada, SPAIN

## Abstract

Two core elements for the coordination of different actions in sport are tactical information and knowledge about tactical situations. The current study describes two experiments to learn about the memory structure and the cognitive processing of tactical information. Experiment 1 investigated the storage and structuring of team-specific tactics in humans’ long-term memory with regard to different expertise levels. Experiment 2 investigated tactical decision-making skills and the corresponding gaze behavior, in presenting participants the identical match situations in a reaction time task. The results showed that more experienced soccer players, in contrast to less experienced soccer players, possess a functionally organized cognitive representation of team-specific tactics in soccer. Moreover, the more experienced soccer players reacted faster in tactical decisions, because they needed less fixations of similar duration as compared to less experienced soccer players. Combined, these experiments offer evidence that a functionally organized memory structure leads to a reaction time and a perceptual advantage in tactical decision-making in soccer. The discussion emphasizes theoretical and applied implications of the current results of the study.

## Introduction

Tactical skills describe the ability of certain players to judge and decide for upcoming game situations appropriately [1]. Tactical knowledge does not just facilitate information processing, but also permits a target-related and purposeful adaptation of behavioral potentials to conditions in the environment [2]. It seems necessary to store and access all relevant information and outcomes of the learning processes in tactical team cooperation as information in long-term memory (LTM) [3]. Thus, an athlete’s performance on the pitch not only involves knowledge about task-specific information, but also a learning-dependent modification of information. The present study investigates expertise-dependent differences in the cognitive representation and the cognitive processing of team-specific tactics in soccer. Despite the fact that tactical skills related to sports performance in ball sports are difficult to access, they have become a fundamental research area. Different tests from psychological research tried to elucidate selected cognitive abilities of soccer-experienced individuals. The Loughborough Intermittent Shuttle Test investigated the mental concentration of soccer players. The authors found that the mental concentration test performance itself did not differ between pre- and post-exercise trials, although these authors focused more on the effects of fluid consumption during soccer [4]. The Stroop Color and Word Tests accessed team sport players’ cognitive functions by evaluating an interference score, which reflected participants’ abilities to resist cognitive interference. In this study, both the color and word scores of team sport players were higher during halftime in comparison to the identical pre-competition test. This elevated halftime score remained constant until the end of the match [5]. Importantly, the transfer of such results to the cognitive processing of team-specific tactics needs further attention. However, the ecological validity of this test as it relates to the cognitive processing of team-specific tactics in soccer players LTM is questionable for complex stimuli such as team-specific tactics in soccer. However, it remains interesting to investigate interfering properties of tactical situations. A better ecological validity exists in the observation and evaluation of soccer matches by video analysis. Early attempts used a recall paradigm (e.g., of player’s positions on the pitch) to evaluate tactical behavior in soccer during the observation of structured video scenes. Less experienced soccer players showed greater recall error of player positions in structured video scenes as compared to more experienced soccer players [6]. More experienced soccer players are able to build up chunks of corresponding information (i.e., meaningful associations between the perceived player positions on the pitch in terms of their tactical goal). Thus, more experienced soccer players are better able to anticipate subsequent match options. They are more successful at anticipating possible passing destinations, because they access contextual information about what happens next and use already acquired knowledge [7]. So far, video analysis techniques in soccer focus on questions regarding the tactical behavior and performance-relevant indicators of the own and the opposing team aiming at the adjustment of the own team’s behavior [8, 9, 10]. Thus, the own team gets aware of repeating game openings, patterns to create shots on goal, or key players in opponent’s playmaking. Importantly, the results of such applied video analysis systems fail to deliver useful information about *the cognitive representation*, *the cognitive processing*, *and the visual information processing* of tactics in soccer [11].

A preferable approach to investigate the cognitive representation of team-specific tactics is by verbal protocol analysis during recall and recognition tests. Specifically, the evaluation of the verbal reports of thinking (i.e., non-structured protocols expressed verbally after the observation of match situations) indicate that more advanced cognitive representations enable more experienced players to retrieve relevant information in order to make appropriate task-specific judgments [12]. A drawback of such methods is their uncertainty about what is exactly measured. Verbal protocols often refer to self-analyses, judgments, or wishes, rather than explicit knowledge or cognitive representations stored in the LTM [13].Furthermore, the *Tactical Skills Inventory for Sports* assessed selected cognitive skills, like positioning and deciding, knowing about ball actions, etc. [14]. That inventory (i.e., in form of a questionnaire) delivered insights regarding the cognitive processing of a few tactical parameters in soccer, which were not related to match strategy. Thus, the integration of the observed tactical parameters towards the choice of players for a particular tactic was missing. Additionally, some authors verified a relation between executive functions and the tactical behavior of Under-15 soccer players [15]. They found differences between low and high performers with regard to the tactical behavior in relation to their affective decision-making skills. Finally, one reasonable hypothesis considered a conceptual organization of run of play structures in the LTM in terms of ‘tactical skills’ [16]. These tactical skills relate closely to representations of tactical problems occurring during sport competitions. It was found that experts, when compared to novice athletes, possess “… more sophisticated conceptual networks of declarative and procedural knowledge (both tactical and motor skill related); procedures for response selection and execution; and specific memory adaptations and structures (e.g., sport specific strategies, situation profiles) that were stored and accessible from LTM” [17].

To learn about soccer players observational strategies it seems promising to investigate their gaze behavior. Therefore, the number of fixations on a presented scene can quantify the gaze control and attention in soccer games. If the number of fixations on an object in a scene is high, than more object properties are perceived, and so much better is the detection of the object functionality [18]. Thus, the number of fixations can be an indicator for the attention towards an object. Roca and colleagues [12] found that experienced soccer players executed more fixations of shorter duration during the decision for an appropriate motor reaction of defense-oriented individual tactics in soccer. Williams, Davids, Burwitz, and Williams [19] found in their study that experienced soccer players responded quicker to open play situations in soccer than inexperienced soccer players. Main difference with regard to the gaze behavior was that inexperienced players fixated more on the ball, and experienced soccer players more on peripheral aspects of the display. Williams and Davids [20] found similar results for 1-on-1 soccer simulations, but not for 3-on-3 simulations. In the 3-on-3 simulation, the experienced players fixated longer on the hip region of the players as compared to inexperienced players. Vaeyens, Lenoir, Williams, and Philippaerts [21] investigated adolescent soccer players and their gaze behavior while passing a ball to one of the teammates. The elite and sub-elite players were better than regional players were, but novice players showed a few parallels in their gaze behavior. The authors Vaeyens, Lenoir, Williams, Mazyn and Philippaerts [22] demonstrated for offense plays that the evaluation of more complex scenarios (e.g., 5 vs 3 or 4 vs. 3 in comparison to 2 vs. 1 or 3 vs. 1) discriminated better between the different expertise levels of the participants. More experienced soccer players shifted their gaze between the player in possession of the ball and other areas as compared to less experienced soccer players. The aforementioned studies in sport tactics revealed that experienced soccer players make faster decisions, and their decisions are of higher quality than those of inexperienced soccer players. Nevertheless, it remains unclear whether this superior decision-making skill bases on the expert’s early detection of relevant cues or on their ability to process the perceived information more effectively. Thus, the current study focusses on the research question, which kind of gaze control enables experienced soccer players to decide for match situations affording selected team-specific tactics.

Overall, differences in gaze behavior may be due to different task constraints [23]. However, differences in the cognitive representation structures, for instance, of the instep kick in soccer lead to different gaze patterns during a decision-making process [24]. Experts, for instance, focused more on the relevant information of the task, which describes a more functional attention [25]. It seems that tactic-related structures in LTM evolve with an increasing level of expertise. Sport and cognitive science researchers recommend that research questions should focus directly on structure formation in the LTM at a tactical level, which influence the behavior [8, 26, 27]. The present study addresses two questions: Are there expertise-related differences between soccer players regarding their cognitive representation of team-specific tactics? Are there expertise-related difference in the gaze behavior while deciding between different team-specific tactics in soccer? We inferred two hypotheses from these research questions. First, in contrast to less experienced soccer players, more experienced soccer players possess a functionally organized representation structure of team-specific tactics in soccer. Second, more experienced soccer players are able to determine an appropriate team-specific tactic faster than less experienced based on their gaze behavior.

## Methods

Two experiments investigate soccer players’ cognitive representation, the cognitive processing, and the visual attention patterns of team-specific tactics in soccer. Both experimental setups used the identical stimulus material.

### Ethics Statement

The participants of both experiments provided written consent prior to the experiment, and received no financial compensation for their participation. The study was conducted in accordance with the ethical principles stated within the declaration of Helsinki (1964), and was approved by the Ethics Committee at Bielefeld University.

### Stimulus Material

**Fig 1:**
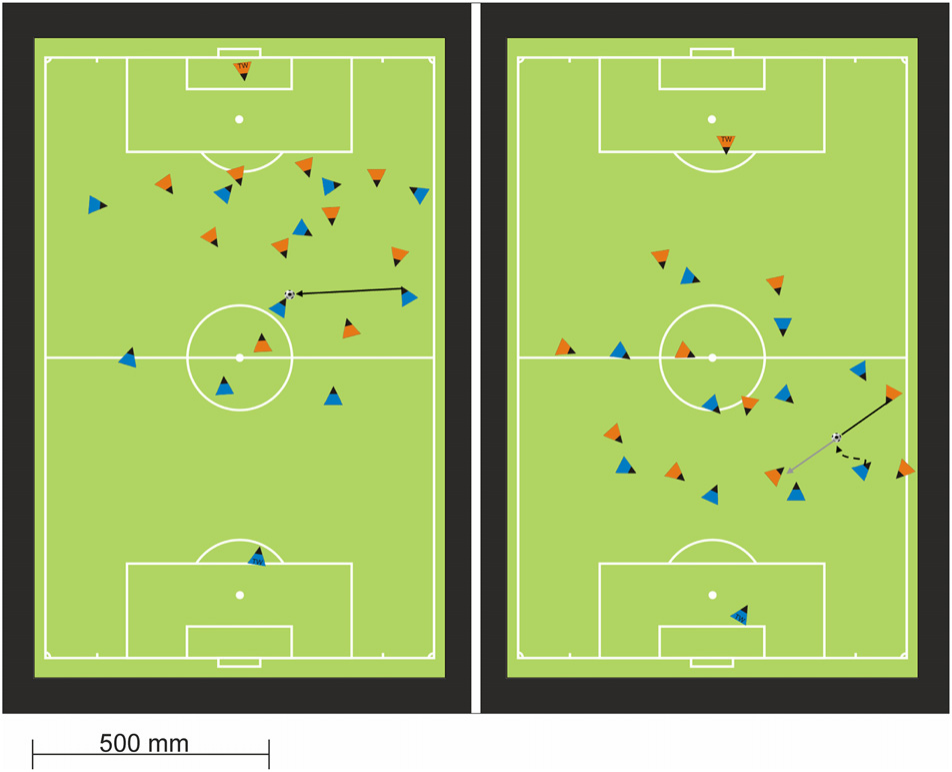
Setup for the Cognitive Measurement of Tactics in Soccer. A projector presented the colored stimuli on a white wall by a beamer. The stimulus on the left side was in anchoring position and compared to every other stimulus. After this procedure, the next randomly chosen stimulus got into the anchoring position to compare it again with every other stimulus.

Four different team-specific tactics in soccer were object of both experiments. These team-specific tactics were (1) counter-attack, (2) change sides, (3) back to defense, and (4) pressing. The team-specific tactics investigated in this study portray a selection (i.e., two in and two without possession of the ball) of all possible team-specific tactics. The match situations were designed in correspondence to the description of fundamental team-specific tactics [28, 29, 30]. Every stimulus portrayed a match situation presented in a coach boards design from a birds-eye perspective. Equilateral triangles depicted all players on the pitch. The orientation of the board was identical throughout the experiments, whereby the participant’s team played offensively in the upward direction indicated by blue triangles, and the opponent’s team played offensively in the downward direction indicated by orange triangles. Fig. 1 shows an exemplarily design of the stimuli. The letters “TW” (German abbreviation for the word goalkeeper) indicated the triangles representing the goalkeepers. A black vertex within each triangle specified the viewing direction of each player. Generally accepted signs and symbols (e.g., solid lines indicated passing directions and dashed lines running paths) provided additional information in the stimulus material. This kind of stimulus design avoids conflicting cognitive processes involved in the perception of body postures, like the perceptual and motor resonance phenomena described by Schütz-Bosbach & Prinz [31], and focusses solely on the cognitive structure formation of tactics.

Three conditions specified each team-specific tactic. First, the global conditions defined parameters like number of players involved, playing direction etc. Second, the specific conditions defined parameters like the player in possession of the ball, the location on the field, the number of players close to ball etc. Third, a situation-specific activator defined an action or event, which directly triggers the specific tactical behavior (e.g., a difficult pass in the back of a defender triggers a pressing behavior). Before the study commenced, an evaluation study determined the relevant match situations to ensure the usage of an appropriate stimulus material. Pre-designed match situations (*N* = 28) were judged by highly experienced coaches (*N* = 8, holding at minimum an A-license from the Deutscher Fußball-Bund and the Union of European Football Associations). These coaches stated, to how many percent (between 0 and 100) the depicted match situation is typical for the afforded team-specific tactic. Coaches’ judgments were inhomogeneous according to the Fleiss Kappa statistic [32] for all raters (κ = 0.263). An Item Fit (IF) calculated by the subtraction of the coefficient of variation multiplied with 100 from the mean integrates the inhomogeneity of the coaches’ judgments in the choice for adequate match situations. The final stimulus set (*n* = 12) emerged from this item fit analysis. Table 1 provides an overview of the Kappa statistics and item fit statistics.

**Table 1:** Overview of Used Stimuli. The stimuli are assigned to the team-specific tactics from participants’ team perspective including a brief description of the scenario. The short description defines broadly the used match situation. The calculation of an item fit based on coaches’ decisions for each stimulus formed the basis to choose three out of seven adequate stimuli for the team-specific tactics applied in the Experiments 1 and 2.

No	Playing direction	Team-specific tactic	Description of match situation	Mean	SD	Item fit
1	Offense	Counter-attack	Steal on the right side in the midfield	80.63	11.48	66.39
2	Offense	Counter-attack	Steal after an opposing corner kick	86.25	23.87	58.58
3	Offense	Counter-attack	Steal in the center of the midfield	75.63	29.45	36.68
4	Defense	Pressing	On the left side in the attacking zone	88.38	08.85	78.36
5	Defense	Pressing	On the right side in the midfield zone	76.13	17.57	53.04
6	Defense	Pressing	On the right side in the attacking zone	77.00	21.02	49.70
7	Offense	Change sides	Shift game play to the left side via the goalkeeper	91.88	12.52	78.25
8	Offense	Change sides	Shift game play to the left side in the midfield	90.00	10.69	78.12
9	Offense	Change sides	Shift game play to the right side in the midfield	89.00	11.25	76.36
10	Defense	Back to defense	After turnover on the left side in the midfield	83.13	23.14	55.29
11	Defense	Back to defense	After turnover in the center in the attacking zone	75.00	18.52	50.31
12	Defense	Back to defense	After turnover on the right side in the midfield	78.13	25.35	45.68

### Experiment 1

Experiment 1 applied the *Structural Dimension Analysis of Mental Representations (SDA-M)*, which implicitly accesses the cognitive representation of complex motor actions in the LTM [33, 34]. The results of the studies provided evidence for a functional organization of cognitive representations in the control of complex human movements. This method reveals the strong relationship between the performance outcome of complex motor actions and the cognitive representation structure. The cognitive representation structure reflects the biomechanical demands of a successful movement execution. However, investigations of the cognitive architecture were not limited to complex motor actions. Additionally, this method revealed differences in the memory structure of children regarding the evaluation of comfortable and uncomfortable grasp postures [35]. Furthermore, the memory structure of general skills such as movement directions has an impact on motor performance [36, 37]. The used stimuli in the mentioned studies (i.e., grasp postures or movement directions) are comparable to the cognitive equivalents of the basic concepts in object categorization described by Mervis and Rosch [38]. The current study investigates the cognitive representation structure in soccer players with regard to their level of expertise. Therefore, Experiment 1 applied match situations depicted as static images, which afford team-specific tactics.

Participants

Experiment 1 investigates two groups of participants. The more experienced soccer players competed at a higher performance level as compared to the less experienced soccer players. The group of less experienced soccer players (*n* = 20) were on average 26.2 (*SD* = 4.2) years old. These players had on average a soccer-specific experience of 3.2 (*SD* = 4.2) years, acquired during (a) club soccer training up to 8^th^ league, (b) university courses, or (c) during non-organized leisure time activities. The group of more experienced soccer players (*n* = 18) were on average 21.8 (*SD* = 2.7) years old. These players had on average a soccer specific experience of 17.3 (*SD* = 3.3) years, acquired during specific soccer training. The more experienced players were members of a team competing in the 4^th^ league in Germany while this study was conducted.

Task & Procedure

The SDA-M measured the cognitive representation of team-specific tactics in soccer. Pictures of three match situations for each of the four team-specific tactics served as stimuli. All participants from the same level of expertise were tested in a single session. Before data acquisition, the experimenter informed all participants about the used stimuli, the coach board’s design, and the used symbols as well as the teams’ playing directions. The experimenter did not address the measurement of team-specific tactics in Experiment 1. A projector displayed the match situations on a white wall (projection size 2 × 2.5 m) to ensure perfect sight for every participant. The projection showed two equally sized parts (see Fig. 1). The left part of the projection presented one randomly chosen stimulus in an anchoring position. The right part of the projection showed one randomly chosen stimulus of the remaining stimuli. The participants indicated whether their team had to react with the same team-specific tactic for each stimulus pair. Participants did not explicitly label or name the underlying team-specific tactics. This is the major difference between the current task and a simple sorting or rating task. This split procedure probes participants’ implicit knowledge base about team-specific tactics in soccer. The decision-making in the split procedure forced participants to detect the underlying tactical behavior without an explicit naming of terms and specifications for the tactics. Participants entered their decisions into a form without temporal constraints. Later, the experimenter transferred their decisions into SDA-M software.

Data analysis

The SDA-M analyzed the cognitive representation of the team-specific tactics in both expertise groups. The analysis of each participant’s representation structure for the twelve different match situations consisted of three steps. In the first step, the participant performed the split procedure (described in Task & Procedure). For each match situation in the anchoring position, the procedure resulted in a positive (i.e., including all match situations requiring the same team-specific tactic according to the participant) and a negative (i.e., including all match situations requiring a different team-specific tactic according to the participant) subset of the remaining match situations. The match situation in the anchoring position was automatically placed into the positive subset. A score was assigned to the match situations in both subsets, which reflected their similarity to the match situation in the anchoring position. The score was calculated as the sign of the subset (positive/negative) times the number of elements within the subset. This procedure resulted in a score vector for each match situation in the anchoring position. The concatenation of all score vectors created a matrix in which each row corresponded to one match situation. A z-normalization of each row converted the score vectors to a relative position of the corresponding match situation in a multidimensional feature space. From this normalized position matrix, a Euclidian distance matrix was calculated. This matrix contained the Euclidean distances between the relative positions of each pair of match situations. The Euclidean distances formed the basis for a hierarchical cluster analysis aiming at a grouping of corresponding match situations.

In the second step, a hierarchical cluster analysis (unweighted average-linkage) was applied to the distance matrix to create the cognitive representation structure of the team-specific tactics in soccer (i.e., a dendrogram). Each dendrogram reflected the cognitive representation structure of team-specific tactics in soccer for a single participant. To calculate the average representation structure of a participant group, the normalized position matrices of all participants are averaged, re-normalized, converted into a Euclidean distance matrix, and subjected to a hierarchical cluster analysis. Based on a critical alpha-level of *p* = .01, a critical Euclidean distance of *d*_*crit*_ = 4.552 was estimated for differences between match situations to be significant. Match situations connected below the critical value form distinct clusters. Conversely, match situations connected above the critical value belong to statistically different clusters.

In the third step, a between-groups invariance measure tested the generated cognitive representations of team-specific tactics in soccer for structural homogeneity. The invariance measure compared resulting dendrograms between groups based on the common number of shared clusters, the common number of match situations within each cluster, and the average quantities of evolved clusters. The measure of the invariance value λ ranges between 0 and 1, whereas 1 indicates the highest accordance between two structures. The statistical threshold for accepting invariance between two structures is set to λ = 0.68 as an empirically estimated value [33, 34, 36, 37, 39]

### Experiment 2

Participants

Two groups of participants had soccer experience with the more experienced players performing on a higher competitive level than the players of the other group. The group of less experienced soccer players (n = 10) were on average 22.7 (SD = 2.0) years old. These players had on average a soccer-specific experience of 0.4 (SD = 1.0) years, acquired during (a) club soccer training up to 7th league, (b) university courses, or (c) during non-organized leisure time activities. The group of more experienced soccer players (n = 10) were on average 25.0 (SD = 3.8) years old. These players had on average a soccer specific experience of 19.8 (SD = 4.4) years, acquired during specific soccer training. The more experienced players received their soccer experience as adults in the 1st and mainly in the 4th league in Germany.

Task & Procedure

Experiment 2 measured participants’ decisions on team-specific tactics within a two-choice reaction time task. Prior to the experiment participants received information about the used symbols and abbreviations in the depicted match situations and the four team-specific tactics. The experimental procedures investigated the cognitive processing of team-specific tactics in soccer.

**Fig 2:**
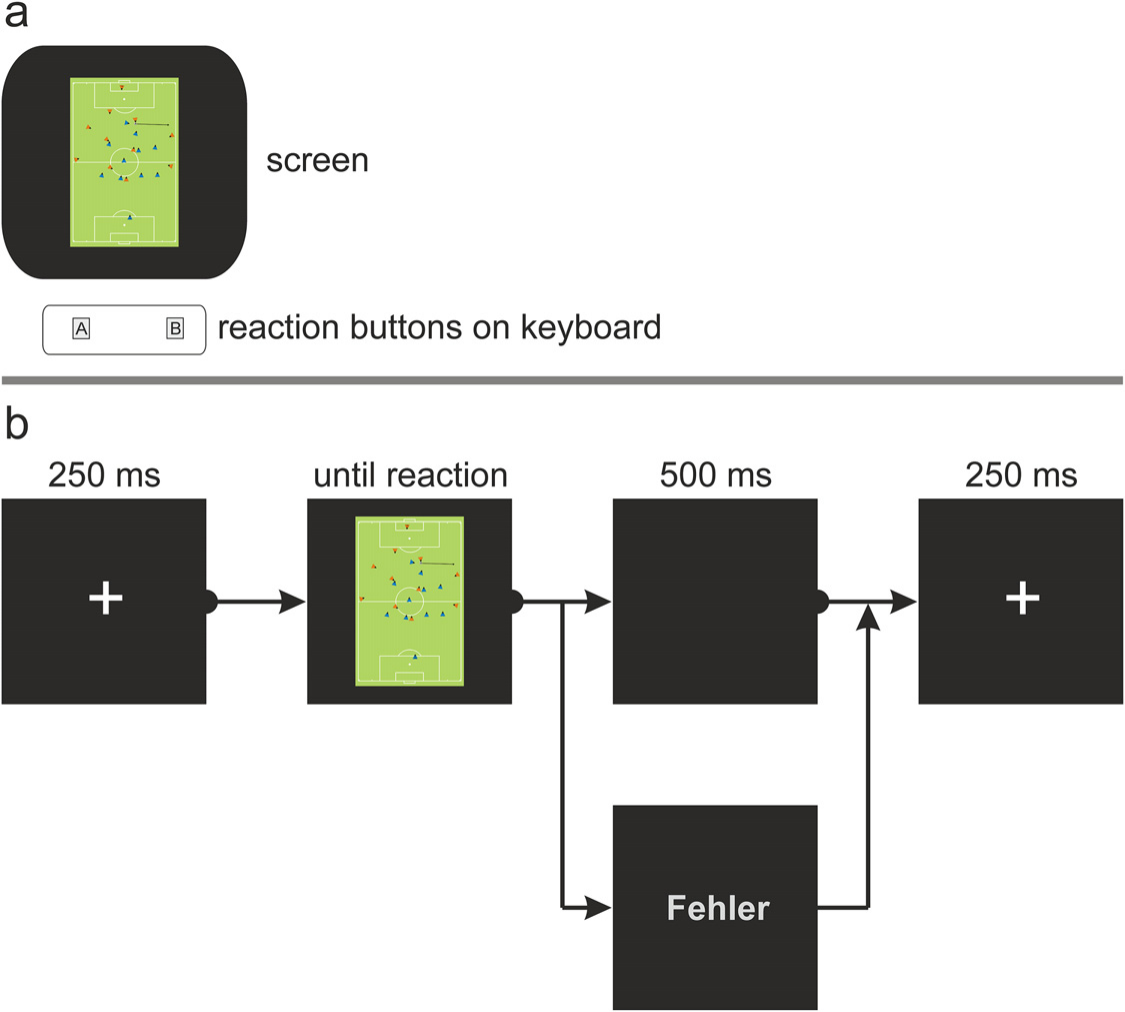
Schematic drawing of the used setup. A screen presented one stimulus at a time. Participants logged their decisions on a keyboard centered in front of the screen by usage of two reaction buttons (each indicating a certain tactical behavior). Each participant completed a test sessions after an introduction of the respective button configurations to ensure they understood the task. (b) Trial sequence of the reaction time task.

The task was for the participants to make a decision between two pre-defined team-specific tactics in the context of one match situation as accurately and as quickly as possible (see drawing in Fig. 2a). Each reaction button was constrained to one team-specific tactic, and the participants were provided with verbal instructions before every practice and test block. The task was to decide whether the presented stimulus belonged to one team-specific tactic or the other. In front of every test block, participants completed a practice block (i.e., six trials) to ensure that participants understood the team-specific tactics, and to make them familiar with the buttons configuration of buttons. Overall, Experiment 2 was conducted in six blocks (i.e., including one practice and one test block each) to cover all potential button configurations: pressing vs. back to defense, counter-attack vs. change sides, pressing vs. counter-attack, pressing vs. change sides, back to defense vs. counter-attack, and back to defense vs. change sides. The order of the presented stimuli was randomized across trials, and the locations of the respective reaction buttons were balanced across participants. A visual programming environment for eye-tracking experiments (i.e., VDesigner) processed the experiment [40].

Each block started with the word “Achtung” (German for “attention”) displayed for 1500 ms to draw participants’ attention towards the monitor and to inform them that the test starts immediately. Fig. 2b presents the procedure. First, a fixation cross was shown at the center of the screen for 250 ms before each stimulus. Second, the presented stimulus remained on the screen until participants pressed one of the two reaction buttons. Immediately after a correct decision, a blank screen was shown for 500 ms and the next fixation-cross appeared. If the decision was incorrect, an error message (i.e., “Fehler”; German for “error”) was displayed for 500 ms before the next fixation cross. The error message provided feedback about participants’ accuracy to remind them of the task.

While participants made their decisions on the presented stimuli (i.e., between stimulus onset and button press) their eye movements were recorded using the SR Research Eye-Tracker. This system employs a headset with two cameras to enable binocular eye movement recording. Further features of the EyeLink II system are a high sampling rate up to 500 Hz and an average on-screen gaze position error between 0.5° and 1.0°. The whole system was calibrated for drift correction every five trials within each block, to minimize the measurement error as much as possible, and to keep the experiment as comfortable as possible for the participants.

Data analysis

The data analysis extracted the number of errors and the corresponding reaction times as well as the parameters number of fixations and fixation duration. Attention maps display the spatial resolution of eye movements by the evaluation of the number of observed pixels within each trial. An attention map shows the activation of each pixel in the observed stimulus with regard to the total time an observer spent on these locations. Attention maps highlight areas within a stimulus receiving high attention by the observer. On the opposite, areas sparsely observed are blurred [41]. The attention map pixel values are ranging between 0.0 (i.e., no attention) to 1.0 (i.e., high attention).

A two-way ANOVA (tactic × group) were used to evaluate the results of both experiments in terms of reaction times, number of fixations, fixation duration, and observed pixels. The within factor *tactic* consisted of four levels (i.e., counter-attack, change sides, pressing, and back to defense), and the between factor *group* consisted of two levels (i.e., more experienced and less experienced soccer players). If Mauchly’s test revealed that the sphericity assumption was violated in the ANOVA, the degrees of freedom were corrected by estimation of sphericity according to the Greenhouse—Geisser correction. The reported effect sizes can be interpreted based on Cohen’s conventions [42] as a small (η^2^ = .01), medium, (η^2^ = .06), or large (η^2^ = .14) effect [43]. The statistics software SPSS 21 conducted the statistical analyses.

## Results

### Experiment 1

**Fig 3:**
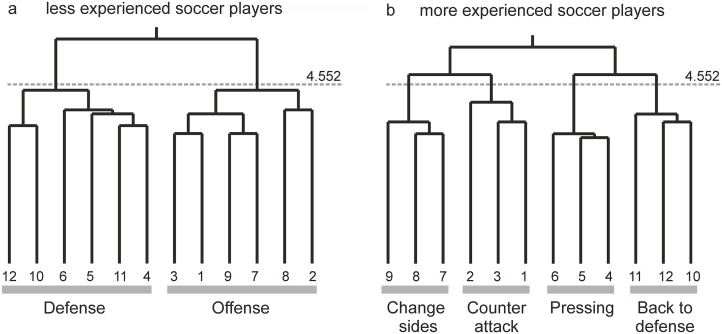
Cognitive representation of team-specific tactics in soccer for less experienced soccer players (a) and more experienced soccer players (b). The number at the bottom represents the different match situations. The height at each conjunction represents the Euclidian distance between match situations. The lower the conjunction, the closer the connection between the match situations. The dashed line represents the critical Euclidian distance, which cut off the branches. The cognitive representation structures show that more experienced soccer players, in contrast to less experienced soccer players, possess a functional representation of team-specific tactics in soccer.

Fig. 3a illustrates the mean cognitive representation structure of the group of less experienced soccer players. Their representation shows two distinct clusters. Cluster 1 (combining the match situations 12, 10, 6, 5, 11, 4) integrates all match situations aligned to defensive tactics, in which the opposing team is in possession of the ball. Cluster 2 (combining the match situations 3, 1, 9, 7, 8, 2) integrates all match situations aligned to offensive tactics, in which the target team is in possession of the ball. There is no further functional clustering in the cognitive representation of team-specific tactics in less experienced soccer players.

Fig. 3b illustrates the mean cognitive representation structure of the group of the more experienced soccer players. Their representation shows a separation of the match situations in four distinct clusters. Cluster 1 (combining the match situations 9, 8, 7) integrates all match situation related to the team-specific tactic ‘change sides’, and Cluster 2 (combining the match situations 2, 3, 1) all situations of ‘counter-attack’. Above the critical Euclidean distance (*d*_*crit*_ = 4.552) these two clusters are merged into the offensive tactics. Cluster 3 (combining the match situations 6, 5, 4) integrates the match situations related to team-specific tactic of ‘pressing’, and Cluster 4 (combining the match situations 11, 12, 10) integrates all match situations related to ‘back to defense’ tactics. The clusters ‘pressing’ and ‘back to defense’ are connected with each other above the critical Euclidean distance (*d*_*crit*_ = 4.552), pointing at the existence of a representation level integrating all defense strategies.

The invariance measure examining for homogeneity between evolved representation structures revealed invariance (λ_more_exp_ = 1.0) of the cognitive representation of team-specific tactics for more experienced soccer players in comparison to an ideal structure. Thus, more experienced soccer players’ cognitive representation can be interpreted as identical to an ideal structure. The cognitive representation of team-specific tactics in soccer for less experienced soccer players revealed no invariance (λ_less_exp_ = 0.42) in comparison to an ideal structure. Both structures share less common features, and thus, they cannot be interpreted as similar to each other.

### Experiment 2

Overall, in 15.3% of all trials, the participants decided incorrectly or data was classified as outlier when decisions were longer than two standard deviations from the mean. ANOVA results for the measured reaction times of the participants correct answers revealed a significant main effect for the factor *group*, *F*(1,18) = 5.486, *p* <. 05, η^2^ = .234. The experienced soccer players (*M* = 1488.2 ms, *SD* = 562.2) decided faster in comparison to the less experienced soccer players (*M* = 2258.9 ms, *SD* = 1121.0). In addition, a significant main effect was observed for the factor *tactic*, *F*(3,54) = 7.694, *p* <. 01, η^2^ = .299. The participants decided faster for the tactic ‘counter attack’ (*M* = 1533.2 ms, *SD* = 733.2), as for ‘change sides’ (*M* = 1558.7 ms, *SD* = 910.4), as for ‘pressing’ (*M* = 2181.8 ms, *SD* = 1059.7), and as for the tactic ‘back to defense’ (*M* = 2220.5 ms, *SD* = 1120.9). There was no significant interaction between the factors, *F*(3,54) = 0.156, *p* = .93, η^2^ = .009.

**Fig 4:**
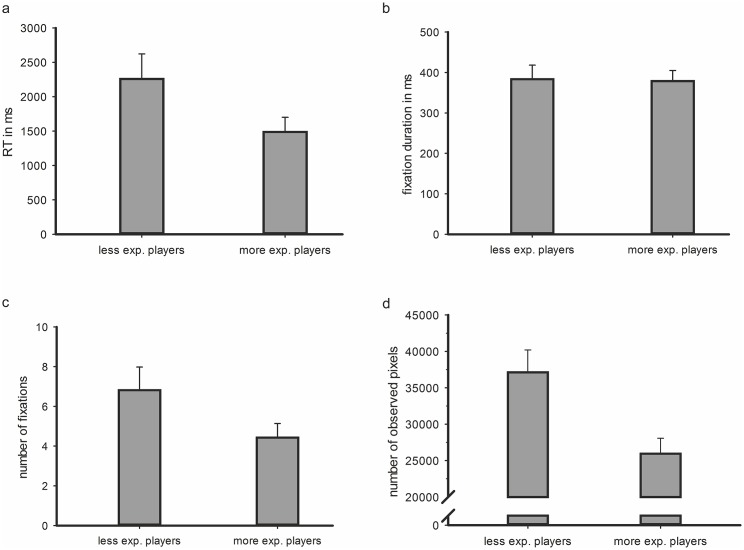
The bar plots show mean results and the error bars (95% confidence interval) of the less and more experienced soccer players. Fig. 4a shows the reaction times, where the more experienced soccer players needed significantly less time to make a correct judgment of the match situation with regard to the appropriate team-specific tactic. Fig. 4b displays the mean fixation duration of the correct decisions. There is no difference between less and more experienced soccer players. Fig. 4c shows the mean number of fixations made between stimulus onset and reaction. The more experienced soccer players needed significantly less fixations as compared to the less experienced soccer players. Fig. 4d indicates the mean number of pixels observed during the decision-making process. The more experienced soccer players observed significantly less pixels within the stimulus material as compared to the less experienced soccer players.

ANOVA results for the average fixation duration of the participants correct answers revealed no significant main effect for the factor *group*, *F*(1,18) = 0.019, *p* = .89, η^2^ = .001. As well the experienced soccer players (*M* = 378.9, *SD* = 75.0) as the less experienced soccer players (*M* = 383.4, *SD* = 104.7) had a similar average fixation duration. However, a significant main effect was observed for the factor *tactic*, *F*(3,54) = 4.170, *p* <. 05, η^2^ = .188. The participants had a smaller average fixation duration for the tactic ‘pressing’ (*M* = 335.8 ms, *SD* = 48.5), as for ‘back to defense’ (*M* = 382.5 ms, *SD* = 81.1), as for ‘counter attack’ (*M* = 400.4 ms, *SD* = 104.7), and as for the tactic ‘change sides’ (*M* = 406.0 ms, *SD* = 122.2). There was no significant interaction between the factors. ANOVA results for the measured number of fixations of the participants correct answers revealed a significant main effect for the factor *group*, *F*(1,18) = 5.031, *p* <. 05, η^2^ = .218. The experienced soccer players (*M* = 4.4, *SD* = 2.0) needed less fixations in comparison to the less experienced soccer players (*M* = 6.8, *SD* = 3.6). In addition, a significant main effect was observed for the factor *tactic*, *F*(3,54) = 4.543, *p* <. 05, η^2^ = .202. The participants needed less fixations for the tactic ‘counter attack’ (*M* = 4.7 ms, *SD* = 2.1), as for ‘change sides’ (*M* = 4.9 ms, *SD* = 3.1), as for ‘back to defense’ (*M* = 6.3 ms, *SD* = 3.8), and as for the tactic ‘pressing’ (*M* = 6.6 ms, *SD* = 3.4). There was no significant interaction between the factors. Fig. 4 summarizes all results.

Moreover, the areas observed by the participants have been analyzed for each match situation. The corresponding attention maps of the correct answers delivered insights regarding the amount and the frequency of observed pixels between stimulus onset and participants reaction. ANOVA results for the number of observed pixel within each match situation revealed a significant main effect for the factor *group*, *F*(1,18) = 17.837, *p <*. 01, η^2^ = .498. The experienced soccer players (*M* = 25945 px, *SD* = 6057) observed less pixels within each match situation than the less experienced soccer players (*M* = 37124 px, *SD* = 9604). Additionally, a significant main effect was observed for the factor *tactic*, *F*(3,54) = 6.568, *p* <. 01, η^2^ = .267. The participants observed less pixels in the match situations of the tactic ‘back to defense’ (*M* = 27955 px, *SD* = 10088), as for ‘counter attack’ (*M* = 29168 px, *SD* = 8477), as for ‘change sides’ (*M* = 34490 px, *SD* = 10798), and as for the tactic ‘pressing’ (*M* = 34524 px, *SD* = 9093). There was no significant interaction between the factors, *F*(3,54) = 0.783, *p* = .51, η^2^ = .042.

**Fig 5:**
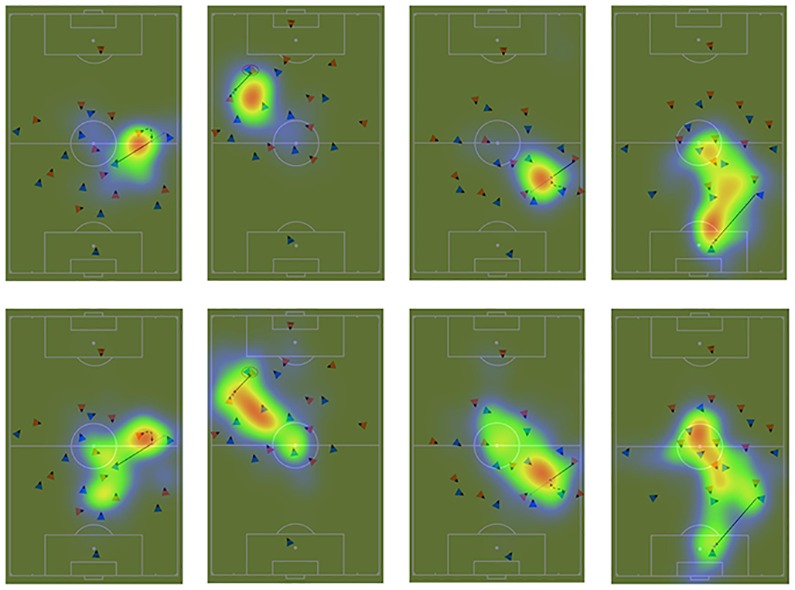
The attention maps for one match situation of each team-specific tactic for the more (upper row) and the less (lower row) experienced soccer players. The match situations represent from left to right one back to defense, one pressing, one counter-attack, and one change sides match situation. Red color indicated areas highly observed, whereas yellow, green, light-green, and blue color indicated areas of less attention in decreasing order. Not attended areas are blurred. More experienced players’ attention is not as distributed as less experienced players’ attention. More experienced soccer players focus on specific areas of the stimuli.

In addition, attention maps visualize the observation strategies. Fig. 5 shows the attention maps of one match situation for each team-specific tactic of both groups. Areas highlighted in red indicate areas, which received high attention. Areas highlighted in yellow, green, and blue indicated sparsely observed areas. Blurred areas where not observed at all during the decision-making.

## Discussion

The current study examined the cognitive representation of team-specific tactics in soccer. The comparative performance of two different groups was examined—a group of more experienced soccer players and a group of less experienced soccer players. The experiments investigated the storage and the cognitive processing of team-specific tactics in soccer in participants’ memory via the cognitive representation structure and a reaction time task. Data supports the hypothesis that more experienced soccer players, as compared to less experienced soccer players possess a hierarchically organized memory structure of team-specific tactics. Less experienced soccer players’ cognitive representation of team-specific tactics in soccer showed a clear separation of tactics related to the playing direction (i.e., offense or defense). In contrast, the more experienced soccer players showed a functional organization of team-specific tactics in soccer that are aligned to the four soccer-specific tactical concepts (counter-attack, pressing, change sides, and back to defense) investigated in this study. These four tactical concepts form separate clusters in the LTM of more experienced soccer players. Independent units within the memory structure represent these team-specific tactics. In addition, more experienced soccer players connect the two defense and two offense tactics at a higher level. These findings suggest that this approach is able to indicate relevant cognitive representations of team-specific tactics in soccer. This extends sport science research by not only documenting specific performance statistics [44], but also by moving beyond such documentation [45]. The present research offers insights into the cognitive representation of team-specific tactics in the LTM, which is difficult to access by the players themselves. Moreover, this parameter is difficult to observe during a soccer match. An observer never exactly knows, whether the perceived behavior is bases on players improved perception, on enhanced physical skills, or on cognitive representation structures. To portray an extensive picture of the performance of soccer players it is necessary to extract and analyze all performance-influencing factors. Thus, the performance indicators like the cognitive representation should form an individual profile, which constitutes ideal athletic performance in comparison to recent performance [27].

Experiment 2 examined the cognitive and perceptual processing of team-specific tactics in soccer. This experiment discovered differences in the behavioral response and observation strategies between less and more experienced soccer players. The results provide evidence that more experienced soccer players needed less time as compared to less experienced soccer players to judge match situations in correspondence to a certain team-specific tactic. The observation strategies during the decision-making process of both groups delivered evidence that mainly the number of fixations necessary to evaluate a match situation is an influencing variable on the reaction time. The mean fixation duration remained constant in both groups. The less experienced soccer players needed more fixations to observe more locations for each match situation, which led to longer reaction times. We assume the hypothesis that more experienced soccer players determine an appropriate tactical behavior faster than less experienced soccer players is correct. In summary, more experienced soccer players classify domain-specific patterns of play faster than less experienced soccer players do. Previous expertise studies already offered evidence for an advanced classification of domain-specific patterns of play (i.e., in terms of recalling more patterns) by experts [46]. Importantly, this has been shown in domains like chess [47, 48], as well as within the sports domain [6, 7, 49]. A likely explanation for this effect is that more experienced players may possess a larger database of chunks, which allows them the faster cognitive processing of such match situations [50]. The present study adds to the knowledge about parameters in decision-making of team-specific tactics in soccer.

Moreover, Experiment 2 delivered evidence that the observation strategies differ between both expertise levels. However, the results suggest that more experienced soccer players do not make more fixations of shorter duration. Instead, the more experienced players needed less fixations of similar duration and observed less pixels in the match situations as compared to less experienced soccer players. This finding contradicts the findings of Roca and colleagues [12]. A potential reason for this discrepancy could be the application of a different stimulus material. Roca and colleagues [12] used video sequences and the present study used static images of coach board designs to indicate the corresponding match situations. Video material contains information about the tactical behavior, but also information about the motor behavior of the involved players. If participants observe the motor behavior, which corresponds to their field of expertise (i.e., soccer) they activate corresponding cognitive representations of complex motor actions. Thus, the brain activity of participants observing an experienced motor action reveals the influence of motor expertise on action observation [31, 51]. Additionally, Williams and Davids [20] already found that more experienced soccer players pay more attention to body postures than less experienced soccer players do. Therefore, the present study used static pictures to focus on the tactical information. In addition, the eye-tracking data of the present study supports the finding of Williams, Davids, Burwitz, and Williams [19] that experienced soccer players show a different pattern of attention distribution (i.e., the more experienced soccer players fixated peripheral targets) during their decision-making as compared to less experienced soccer players. Vaeyens, Lenoir, Williams, and Philippaerts [21] showed that successful decision makers in soccer spend more time in fixating the player in possession of the ball and shifted their gaze more frequently between that player and other areas of the stimulus. This finding is contradictory to Roca and colleagues [12] and the results of the present study. The reason might be that Vaeyens, Lenoir, Williams, and Philippaerts [21] used only group specific tactics involving not more than eight players. In addition, participants viewing angle was from a central midfielder in only offense plays. Such restrictions might trigger a special gaze behavior, especially when the participants had to imagine themselves as the midfielder indicated in the video sequences. The data of Vaeyens, Lenoir, Williams, Mazyn and colleagues [22] delivered evidence that with an increasing complexity of the stimulus material (i.e., involvement of players from 3 up to 8) the number of fixations increased. Thus, a higher amount of fixations enables soccer players to perceive the relevant information of all 22 players displayed in the stimuli.

Based on the data of the current study it seems plausible that cognitive representations of team-specific tactics control the gaze behavior in soccer in a comparable manner to the gaze behavior in the perception of complex motor actions [52]. More experienced soccer players needed less fixations to evaluate the underlying tactics, because they possess a functional knowledge of the team-specific tactics. Thus, they were able to make faster decisions, because they identified the most informative locations. The attention maps visualize the spatial distribution of attention across the different match situations. More experienced soccer players focused on selected spots within each match situation. Thus, the inspected areas are smaller as compared to less experienced soccer players. Taken more experienced soccer players number of fixations and the fixation duration into account it seems that they exactly know where relevant cues for a proper decision-making are in the match situations. That enables more experienced soccer players to decide faster. These findings add to the existing knowledge that experienced soccer players are better able to evaluate and prioritize a possible individual tactical behavior (e.g., passing options) than novices [7].

Interestingly the reaction times were fastest for the offense tactics where participants needed as well less fixations to judge them in comparison to the defense tactics. Moreover, the fixation duration was shorter for the defense tactics as compared to the offense tactics. This observation leads to the assumption of a different processing of offense and defense tactics. This finding supports the results from Brazilian soccer [53]. Moura and colleagues [53] found that the area spanned between players in offense is larger than in defense situations. It seems plausible that the viewing strategy for defense situations (i.e., shorter and more fixations) depends on a constant observation of the distances between players. Short distances between players characterize defense situations, because that offers the opportunity to support each other. In contrast, the viewing strategy in offense situations (i.e., less and longer fixations) depends on the observation of certain points of interest (e.g., where to pass the ball). To access relevant parameters for the decision soccer players tend to observe these areas are precisely to avoid a turnover. However, separate studies need to investigate such a phenomenon.

From an applied perspective, the results of the present study suggest practical training implications. If an athlete’s memory structure has an influence on the time it takes to decide for an appropriate tactic, then coaches are able to address players’ cognitive representation explicitly within training sessions. That is what coaches are already attempting, albeit without verified knowledge about their athletes’ memory structures. Thus, the measurement of cognitive representations of team-specific tactics in soccer possess the potential to meet the criteria of a diagnostic tool, which is able to predict future behavior [54, 55]. Coaches are able to address specific tactical problems of athletes within their tactical training. The measurement of cognitive representations supports already training scenarios in Judo (e.g., a Judo throwing technique called Uchi-Mata). High-level experts in Judo possess inter-individual differences in their cognitive representation structure. Practical training implications used such information [56].

Nevertheless, there are some limitations of the current study. First, it seems obvious to increase the ecological validity of the used stimulus material to a comparable match environment in soccer. That means that the stimuli should be real video sequences of soccer matches. To infer better to the discrete behavior of players on the pitch they need to observe a match situation similar to their perspective during a match, and not from a bird’s eye perspective as used in the current study. Second, the static stimuli lack information about the dynamical parameters of a match situation. Therefore, video sequences provide more information about where the ball exactly is at which moment in time. The usage of video sequences might solve this problem as well. However, the usage of video sequences increase the ecologic validity, but includes the perception of the motor behavior of actors, which infer with the results of the gaze behavior. A possible solution deliver the application of a virtual reality scenario. It seems possible to use human-like dummies, which replace humans in the match situation. Therefore, the displayed match situation preserves all dynamical aspects and excludes the motor behavior. Third, other tactical behaviors (i.e., group-specific or individual tactics) are of similar relevance for the soccer-specific performance. Thus, following studies should focus on such tactical behaviors.

Moreover, the proposed methods in the present paper investigated soccer players’ cognitive representations and the cognitive processing of team-specific tactics in soccer. The results of the studies facilitate the understanding of soccer experts’ tactical decision-making as proposed similarly by Vestberg, Gustafson, Maurex, Ingvar, and Petrovic [57]. In addition, the knowledge about the most informative locations during the decision-making process may facilitate the learning. The presented results deliver evidence for a superior decision-making skill of experts related to an early cue perception and not to an advanced processing of fixated information. Thus, the evaluation of cognitive representations of team-specific tactics in soccer in combination with the measurement of gaze behavior has the potential to form the basis for the diagnostics in and the development of functional tactics training. Especially, the visual perception can be assessed online during soccer games (i.e., by the usage of new eye-tracking hardware) to evolve visual and mental guided training techniques.

The significance of this study is evident in two perspectives. First, from a theoretical point of view, the cognitive representation and cognitive processing of team-specific tactics is a crucial ability within the performance determining skills in soccer. Second, from an applied point of view, the knowledge about the individual cognitive representation of team-specific tactics in soccer has the potential to plan specially designed training sessions for athletes, and facilitates the individual learning processes.
